# How does precursor RNA structure influence RNA processing and gene expression?

**DOI:** 10.1042/BSR20220149

**Published:** 2023-03-01

**Authors:** Austin Herbert, Abigail Hatfield, Lela Lackey

**Affiliations:** Department of Genetics and Biochemistry, Clemson University Center for Human Genetics, Greenwood, SC, U.S.A.

**Keywords:** alternative splicing, polyadenylation, precursor RNA, RNA structure, splicing, transcription

## Abstract

RNA is a fundamental biomolecule that has many purposes within cells. Due to its single-stranded and flexible nature, RNA naturally folds into complex and dynamic structures. Recent technological and computational advances have produced an explosion of RNA structural data. Many RNA structures have regulatory and functional properties. Studying the structure of nascent RNAs is particularly challenging due to their low abundance and long length, but their structures are important because they can influence RNA processing. Precursor RNA processing is a nexus of pathways that determines mature isoform composition and that controls gene expression. In this review, we examine what is known about human nascent RNA structure and the influence of RNA structure on processing of precursor RNAs. These known structures provide examples of how other nascent RNAs may be structured and show how novel RNA structures may influence RNA processing including splicing and polyadenylation. RNA structures can be targeted therapeutically to treat disease.

## Introduction

### Are precursor RNAs structured?

The sequence of an RNA influences its biological activity. Sequence information still predominates as the most studied aspect of a nucleic acid. However, due to its single-stranded and flexible nature, RNA naturally forms structures as soon as it is synthesized by an RNA polymerase, reviewed in [1,2]. There is clear evidence for robust and reproducible RNA structures in RNA transcripts, both in human cells and several model organisms [3–8]. RNA structures can range from stable, consistent folds to flexible structural ensembles, reviewed in [9]. Transcriptome-wide structure analysis has revealed patterns of functional structure in processed RNAs, including flexible structural transitions around start and stop codons [3,5,6,8]. However, little is known about RNA structure in nascent human RNAs before they undergo processing to become mature transcripts. There is evidence that RNA structure is altered at different stages of an RNA molecule’s life cycle. Liu et al. found major differences between nuclear and cytoplasmic RNA structures in *Arabidopsis*, which suggested that RNA structures changed significantly from the nascent RNA to the mature transcript [10]. Structures in 3′UTRs can vary during different stages of development in zebrafish [11]. In yeast, there are differences in structure between the same RNAs *in vivo* versus RNAs extracted from the cell [4]. Direct study of nascent RNA structure is important to understand the relationship between precursor and mature RNA structures and how precursor RNA structure can impact RNA processing and gene expression. Although still limited, several studies have identified transcriptome-wide patterns of RNA structures in nascent RNAs [10,12].

### What methods are available to study nascent RNA structures?

Experimentally based secondary structural models of RNAs can be created with enzyme and chemical probing data, reviewed in [13]. In particular, chemical probing combined with next-generation sequencing has become very popular due to its ability to generate data on long RNAs and multiple RNAs at the same time [14,15]. Chemical probes include those that react with the ribose backbone (selective 2′-hydroxyl acylation analyzed by primer extension (SHAPE) reagents) and those that react with nucleobases, such as dimethyl sulfate and carbodiimides [16,17]. There are a wide variety of chemical probing applications (Table 1). While chemical probing primarily reveals secondary structure, there are other structure modeling techniques capable of measuring tertiary structures including cryo-electron microscopy (cryo-EM), nuclear magnetic resonance (NMR) and small angle X-ray scattering (SAXS). Recent advances in cryo-EM technology have resulted in structures of large RNA and protein (RNP) complexes like the spliceosome and ribosome [18,19], suggesting that deriving tertiary structural models for stable structures in large pre-processed RNAs may be possible. Secondary and tertiary RNA structural models can be modelled computationally and incorporate experimental chemical probing data [20]. These calculated structures can be based on combinations of thermodynamic parameters, machine learning and experiments, including highly cited methods like RNAstructure, mFOLD and others [21–27]. However, though less time consuming than experimentally generated structures, the accuracy of *de novo* models of RNA structure, both secondary and tertiary, is questionable [28–30].

**Table 1:** Chemical probing methods

Protocol	Brief description	Examples	Pros	Cons
Reverse transcriptase (RT)-stop	During reverse transcription chemical adducts cause RT fall off. Truncated products are run on a gel.	DMS RT-Stop [179,180]	Capable of measuring nucleotide accessibility in flexible RNAs	Restricted to short sequences, one RNA at a time, does not determine specific base-pairs
MaP (mutational profiling)	During reverse transcription chemical adducts are replaced with mutations and read out by sequencing.	DMS-MaP [14], SHAPE-Map [15,181]	Capable of measuring accessibility in long, flexible RNAs and multiple RNAs at the same time	Requires high read depth, does not determine specific base pairs
RNA pulldown	The chemical probe is tagged (i.e., click chemistry, biotin-conjugation, etc.). RNAs with adducts are enriched.	icSeq [8], SHAPES [182]	Captures low abundance RNAs by enrichment	Enrichment may disrupt reactivity calculations, does not determine specific base pairs
Protein immunoprecipitation	Probed RNAs are co-precipitated by a protein-targeted antibody for analysis.	tNET-Structure-Seq [12], fSHAPE [183,184]	Specifically targets RNAs associated with a protein	Requires high read depth, depends on antibody specificity and affinity, does not determine specific base pairs
Hybridization-capture	Probed RNAs are targeted by tagged oligonucleotides (i.e., biotinylated-U) for analysis.	SHAPE-MaP enrichment [31]	Can measure accessibility in low abundance RNAs	Requires production of oligonucleotides to target RNAs, does not determine specific base pairs
Cross-linking	Nucleotides in close proximity are covalently linked by UV and/or crosslinking compounds. The RNA is enriched, cleaved, and ligated for junction analysis.	PARIS [185], CLASH [186], SPLASH [187], LigR-Seq [188], RIC-Seq [189]	Identifies long-distance base pairing, determines specific base pairs	Can have false positives, requires high read depth
Selection by 3′ end sequencing	Probed RNAs undergo 3′ sequencing using polyA oligo hybridization, cleavage and polyA priming.	DIM-2P-Seq [60]	Improves structure definition at the 3′ end of transcripts	Requires high read depth, does not determine specific base pairs

Listed techniques are based on chemical probing where RNA is treated with a chemical that typically forms adducts with accessible nucleotides. The modified RNA is converted to DNA by reverse transcription.

A major hurdle in acquiring experimental secondary structure data from *in vivo* systems is that most methods depend on a significant number of molecules to quantify reactivity (Table 1, Cons). Thus, current *in vivo* methods are limited to high abundance transcripts. Low abundance transcripts that do not meet copy number thresholds require *in vitro* or more elaborate techniques to deduce structure [31,32]. The low abundance of precursor RNAs is one reason that their structures are understudied. Some techniques are beginning to address the problem of low abundance RNAs, including enrichment for low abundance targets during chemical probing (Table 1, RNA pull-down and Hybridization capture) [31,33,34]. Another hurdle in studying the structure of precursor RNAs is the flexible nature of RNA molecules. Although some RNAs have stable structures, such as transfer RNAs and RNAs found in the spliceosome and ribosome, most RNAs have many possible structures that are energetically similar. This ensemble effect of RNAs must be considered when determining what structures are biologically relevant. The long length of most introns compounds this problem for precursor RNAs. Computational modeling to analyze ensembles is being applied to assist in this problem [35–37]. Additionally, many RNAs are likely to have long-distance and tertiary interactions that may be functional but remain difficult to map [38,39]. Little is known about the importance of long-distance interactions in pre-processed RNAs. Experimental mapping techniques are available to document long-distance interactions (Table 1, Cross-linking). Finally, the nature of co-transcriptional processing adds a temporal element to precursor RNA structure modeling. Introns can be spliced out of order, making it difficult to predict which nucleotides are available for structural interactions during transcription of an RNA [40–42]. There are experimental and computational approaches that partially address the temporal nature of RNA folding by targeting temporally associated proteins (Table 1, Protein immunoprecipitation) [12,43]. However, the specialized approaches that address the problems of RNA structure modeling in dynamic, long and low abundance RNAs are not easily broadly applied.

### Are structures in precursor RNAs functional?

**Figure 1:**
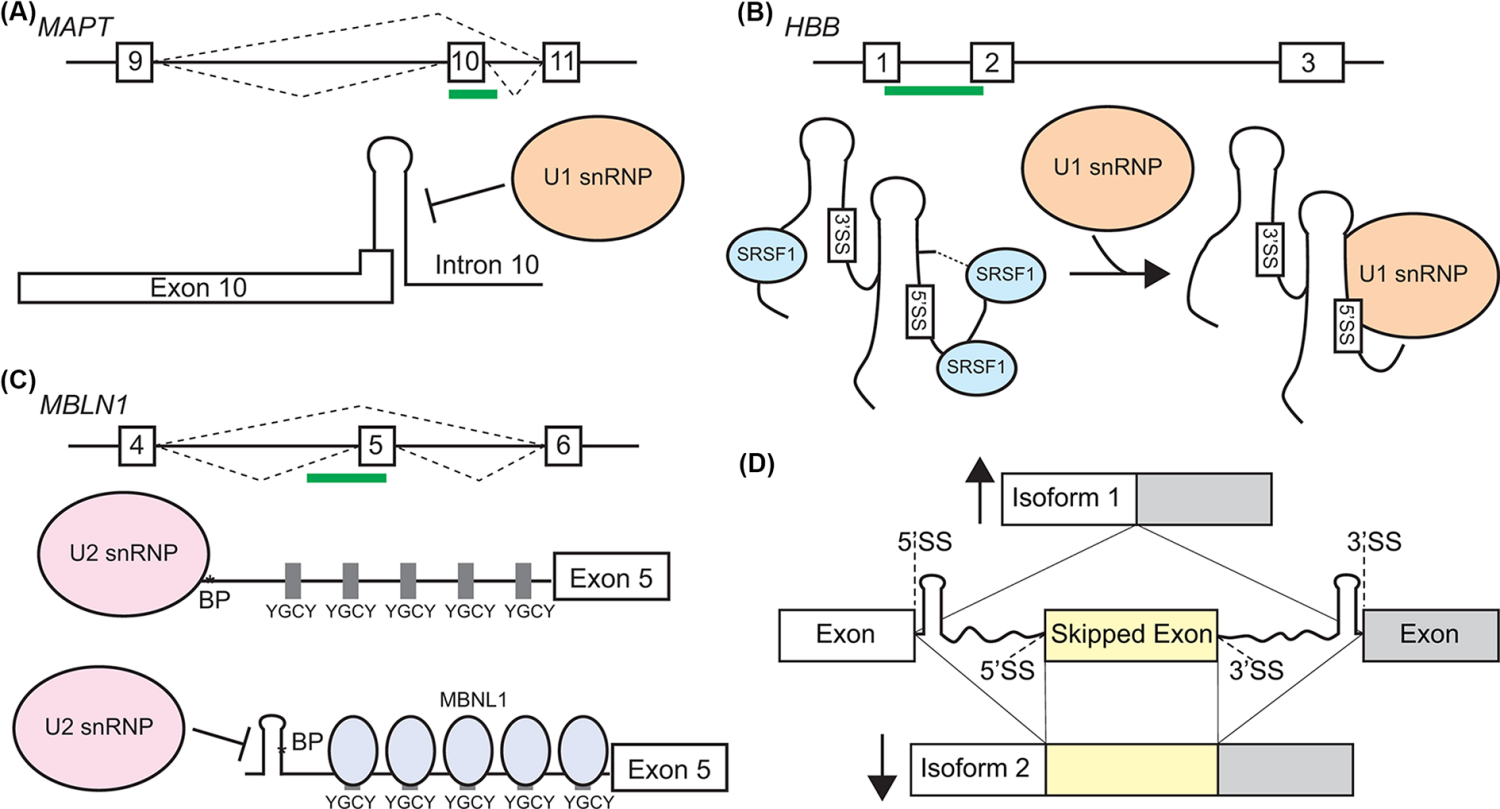
RNA structures influence precursor RNA processing (**A**) Hairpin elements can block 5′ splice site recognition by interfering with U1 snRNP binding. *MAPT1* RNA exon 10 alternative splicing is controlled by hairpin structure at the 5′ splice site. (**B**) RNA structure can bring distal elements in close proximity. The global fold of the *HBB* RNA is mediated by SRSF1 binding and orients the 5′ and 3′ splice sites for U1 snRNP interaction and efficient splicing. (**C**) Recognition of RNA elements are control processing. MBNL1 protein binds to its own RNA. MBNL1 binding causes remodeling of the RNA structure around the branchpoint and represses exon 5 inclusion. (**D**) Transcriptome-wide structural analysis of nascent RNA found clear structural ‘steps’ in proximity to efficiently spliced exons (top). The structure ‘steps’ around frequently skipped exon are less evident (bottom). Cartoon depiction based on [12].

There is no self-evident reason to assume that the structures that RNA molecules form are inherently functional. However, careful research has identified many functional RNA structures and their mechanisms. RNA structures can act to block recognition motifs. Short hairpin motifs that block recognition of the 5′ splice site are common and one of the earliest known structures to functionally impact splicing [44,45], reviewed in [46]. For example, the nascent *MAPT* (*microtubule associated protein tau*) RNA has a hairpin element at the 5′ splice site of exon 10 (Figure 1A). *MAPT* is important in neural biology and its precursor RNA is alternatively spliced to create at least 6 isoforms, reviewed in [47]. A splice junction in *MAPT* (exon 10–intron 10) is normally spliced at an equal ratio, resulting in mix of mature isoforms with either 3 or 4 microtubule binding domain repeats, which code for Tau proteins with different biological activity [48]. A hairpin at the *MAPT* exon10–intron10 junction directly overlaps with the 5′ splice site and can be disrupted by disease-associated variants [49,50]. The *MAPT* hairpin blocks normal spliceosomal recognition by the U1 snRNP at the 5′ splice site and causes exon skipping and formation of the shorter 3R *MAPT* mature transcript [51] (Figure 1A). RNA structure in pre-processed transcripts has been shown to block U1 interactions and alter splicing in other precursor RNAs in addition to *MAPT*, including *SMN2, VWF, ATM* and *BCL2L1* [52–56] (Table 2).

**Table 2:** Mammalian genes containing functional RNA structures that affect RNA processing

Gene symbol	Gene name	Impact	Mechanism	Citations
**MAPT**	Microtubule associated protein tau	Splicing	Potential to block U1 snRNP binding	[50,51]
**XBP1**	X-box binding protein 1	Splicing	Recognition by IRE1	[12,190]
**H2AC11, Histones**	H2A clustered histone 11	3′ end cleavage	Recognition by SLBP	[129]
**PLEC**	Plectin	Splicing	Recognition by SNRPA1	[61]
**TNNT2**	Troponin T2, cardiac type	Splicing	MBNL1 and U2AF65 competition	[63,191]
**MBNL1**	Muscleblind-like splicing regulator 1	Splicing	Recognition by MBNL1	[31,62]
**FN1**	Fibronectin 1	Splicing	Recognition by SR proteins	[56]
**SMN2**	Surviver of motor neuron 2, centromeric	Splicing, polyadenylation	Block U1 snRNP binding, PIE element	[55,90,119]
**VWF**	von Willebrand factor	Splicing	Potential to block U1 snRNP binding	[53]
**ATM**	ATM serine/threonine kinase	Splicing	Potential to block U1 snRNP binding	[192]
**CFTR**	CF transmembrane conductance regulator	Splicing	Potential to interfere with U6 snRNP	[192]
**TERT**	Telomerase reverse transcriptase	Splicing	G-quadruplex RNA	[193,194]
**TP53**	Tumor protein p53	Splicing	G-quadruplex RNA	[195]
**BCL2L1**	BCL2 like 1	Splicing	G-quadruplex RNA and U1 blocking hairpin	[52,55]
**FMR1**	Fragile X messenger ribonucleoprotein 1	Splicing	FMR protein G-quadruplex binding	[196]
**CD44**	CD44 molecule (Indian blood group)	Splicing	G-quadruplex RNA	[197]
**ENAH**	ENAH actin regulator	Splicing	Long-distance pairing blocks RBFOX binding	[198,38]
**DST**	Dystonin	Splicing	Long-distance pairing	[38]
**PLP1**	Proteolipid protein 1	Splicing	Long-distance pairing	[199,38]
**SF1**	Splicing factor 1	Splicing	Long-distance pairing	[38]
**DNM1**	Dynamin 1	Splicing	Long-distance pairing	[38]
**ATE1**	Arginyltransferase 1	Splicing	Long-distance pairing	[38,200]
**PSEN2**	Presenilin 2	Splicing	Unknown	[201]
**FGB**	Fibrinogen β chain	Splicing	Promotes TRA2B binding and splicing	[202]
**CENPB**	Centromere protein B	Polyadenylation	Collapsed distance between polyA site and cleavage site	[60]
**U1A**	U1 small nuclear ribonucleoprotein A	Polyadenylation	PIE element	[118,155]

RNA structure can also function to collapse the distance within long sequences to bring RNA elements into close proximity. Some nascent RNAs may use structure to condense long intronic regions to form structures that are suitable scaffolds for early spliceosome recognition and activation. The human *AdML (adenovirus 2 major late transcript IVS1*) precursor RNA has a global fold important for splicing [57]. Disrupting the structure of pre-processed *AdML* RNA prevents it from being recognized efficiently by the U1 snRNP in *in vitro* studies. FRET analysis confirms that the 5′ and 3′ splice sites are in close proximity in the normal structure, but not in poorly spliced mutants with altered structures [57]. In the *AdML* precursor RNA the global fold is not dependent on proteins. Similar studies of the human *HBB (β globin*) precursor RNA support the role of global RNA structure in recruiting U1 and promoting splicing [58] (Figure 1B). However, the global fold of the pre-processed *HBB* RNA is influenced by binding of SRSF1 protein as a structural stabilizing factor (Figure 1B). The ability of RNA structure to influence splicing by collapsing the distance between the branchpoint and the 3′ splice site has been documented in several introns in yeast [59]. RNA structure may also collapse the distance between the polyadenylation recognition motif and the cleavage site during 3′ processing and polyadenylation of nascent RNAs [60].

RNA structures can be recognized specifically, often in combination with sequence elements. Small nuclear ribonucleoprotein polypeptide A′ (SNRPA1) recognizes intronic sequence-independent stem structures combined with sequence-dependent loops to promote splicing of cassette exons in multiple genes, including *plectin* (*PLEC*) precursor RNA [61]. SNRPA1 splicing of *PLEC* contributes to a prometastatic cellular environment and is associated with progression and poor prognosis in breast cancer [61]. *MBLN1* precursor RNA is autoregulated by MBLN1 protein binding at primarily unpaired YGCY motifs close to the 3′ splice site [62] (Figure 1C). Binding of MBLN1 to *MBLN1* RNA restructures a distal branchpoint and results in exon 5 skipping and an isoform of MBLN1 protein with different subcellular localization [31] (Figure 1C). RNA structural elements can utilize multiple attributes of folding. In addition to containing structures that specifically bind MBLN1, the global fold of the *MBNL1* exon 5 has also been shown to bring the 3′ and 5′ splice sites into close proximity [31,62]. MBLN1 protein also binds to loop elements with YGCY sequences in other nascent RNAs, including cardiac troponin RNA (Table 2) [63].

Despite technical difficulties in performing transcriptome-wide studies of precursor RNA structure, recent studies have broadly analyzed precursor RNA structure to identify global patterns of functional base pairing. Saldi et al. found that there are higher-order structural ‘steps’ that demarcate efficiently spliced and structured introns from less efficiently spliced exons in human cells [12]. The researchers used tNET-Structure-Seq to acquire structural data on nascent RNAs in human cell lines. tNET-Structure-Seq combines enzymatic and chemical structure probing with RNA Polymerase II immunoprecipitation to determine the accessibility of nascent RNA nucleotides. Introns spliced co-transcriptionally were associated with clearer structural ‘steps’ at the 5′ and 3′ splice sites when compared with splice junctions in introns that were spliced post-transcriptionally [12]. Structural ‘steps’ are characterized by a disparity in nucleotide accessibility around the exon–intron junction. Typical structural ‘steps’ have higher accessibility on the exonic side of the junction and lower accessibility on the intronic side of the junction (Figure 1D). The magnitude of a structural ‘step’ also leads to splicing preferences in cassette exons with bigger differences between the accessibility of the exon and intron leading to more efficient splicing. For example, mutually exclusive exons, a type of cassette exon, are primarily spliced post-transcriptionally and exon-inclusion or exclusion is influenced by RNA structure. Excluded exons had more robust ‘steps’ at the farthest 3′ splice site, whereas included exons were more likely to have weak ‘steps’ at the farthest 3′ splice site [12]. These finding are consistent with the general lack of base-pairing at the 3′ splice site and structural differences based on splicing efficacy found in *Arabidopsis* seedlings [10,64]. The pattern of unpaired, accessible exonic nucleotides and paired intronic nucleotides in efficiently spliced transcripts has also been found in mouse precursor RNAs [65] and other organisms [31,57,64–67], reviewed by [68,69].

## Approaches to identifying functional structures

### How can we distinguish functional structures in a sea of RNA structure?

**Figure 2:**
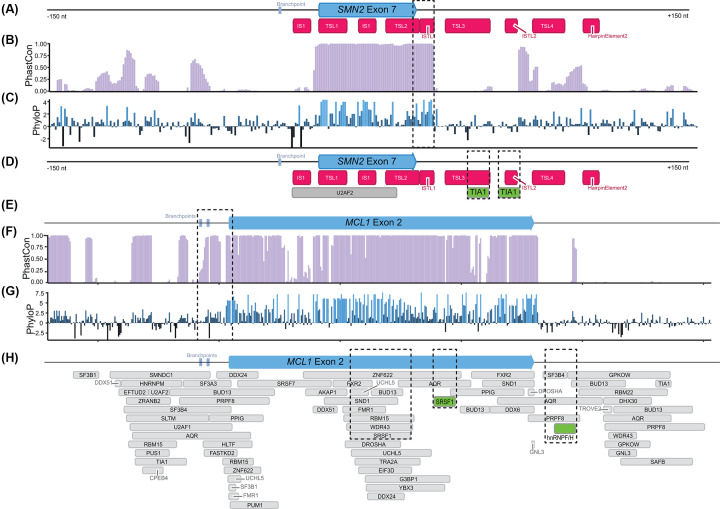
Identifying potential functional structures in *MCL1* RNA using nucleotide conservation and protein binding sites (**A**) Schematic of human *SMN2* exon 7 surrounded by 150 intronic nucleotides on both sides. Known RNA structures in *SMN2* are annotated (red). (**B** and **C**) Nucleotide conservation in *SMN2*. Higher values indicate more conservation. A region of high conservation extends into the 5′ splice site of exon 7 and overlaps with TSL2 and ISTL1 (boxed). (**D**) Protein binding sites across *SMN2* from ENCODE (gray) and published studies (green) are mapped on to the schematic. Binding sites for the RBP TIA1 overlap with TSL3 and ISTL2 structures (boxed). (**E**) Schematic of human *MCL1* exon 2 surrounded by 150 intronic nucleotides on both sides. (**F,G**) Nucleotide conservation in *SMN2*. Higher values indicate more conservation. A region of high conservation extends into the 3′ splice site of exon 2 and overlaps with the branchpoint region (boxed). (**H**) Protein binding sites across *MCL1* from ENCODE (gray) and published studies (green). Binding sites for regulatory RBPs, SRSF1 and hnRNPF/H are indicated (boxes). For both RNAs, schematics were visualized with Geneious Prime v2022.1.1 [203]. Branchpoints were annotated based on experimental data [80]. Conservation data were retrieved from UCSC table browser (Cons 100 Verts, phastCon, phyloP100way) [79]. ENCODE eCLIP data were retrieved as bigBed narrowPeak annotations filtered by a *P*-value of < 0.05, and annotations collapsed for overlapping peaks of the same protein [85]. All data reference hg38.

All RNAs have structural patterns, many of which are functionally important, yet it can be difficult to identify which RNA structures are relevant without prior knowledge. Unlike many nascent RNAs, the survival of motor neuron 2 (*SMN2*) precursor RNA has been extensively mapped for structural elements [55,70–72], reviewed in [73]. *SMN2* splicing studies have focused on exon 7, which can either be skipped or included. Two terminal stem-loops (TSL1 and TSL2) within exon 7 and several structures within intron 7 influence splicing (Figure 2A) [55,70,71,74]. These structures block U1 recognition of the exon 7 at the 5′ splice site and influence RNA-binding protein (RBP) interactions with enhancer and silencer functions. Inclusion of exon 7 allows *SMN2* mature transcripts to produce active SMN protein and promote neural survival in the event of defects at the *SMN1* locus [75,76]. Similar to exon 7 in *SMN2*, exon 3 of *MCL1* can be skipped or included, resulting in the production of short (*MCL1-S*) and long (*MCL1-L*) RNA and protein isoforms with either pro- or anti-apoptotic functions [77]. In contrast with the extensive research into *SMN2* RNA splicing, very little is known about how RNA structure influences RNA processing in most other nascent RNAs, including *MCL1*. We use *SMN2* and *MCL1* nascent RNAs as examples to discuss how to identify functional nascent RNA structures for validation.

One approach to identifying functional regions that may have important RNA structures is to take advantage of conservation data [78,79]. Highly conserved regions within a gene may have functional importance. Both *SMN2* (Figure 2B,C) and *MCL1* (Figure 2F,G) follow a typical conservation pattern with higher conservation values in the exonic regions and lower conservation in the intronic sequences. A region of SMN2 that stands out is the continuation of high conservation scores into the intronic region of intron 7 (Figure 2B,C, boxed). This region overlaps with TSL2 and internal stem loop 1 (ISL1), which sequester the 5′ splice site and promote exon 7 skipping [55]. Additional conserved regions overlap with the functional long-distance internal stem 2 (ISTL2) [71] and TSL4. In *MCL1*, high conservation values are also extended into intron 2, overlapping with the 3′ splice site and experimentally identified branchpoints [80] (Figure 2F,G, boxed). In addition to standard conservation data, covariation analysis can be used to identify functional structures by looking for regions where base pairing is conserved [81,82]. Covariation has been important for identifying functional structures in lncRNAs; however, it is not commonly detected in human protein coding RNAs [83,84]. Conservation data is not structure-specific, however, in *SMN2* conservation data indicates important structural regions, suggesting that highly conserved regions in *MCL1* may have structural significance.

Many nascent RNA processing steps rely on RBP interactions. Mapping RBP-binding sites onto RNA can highlight important regulatory regions. Structures nearby or that overlap with RBP sites may influence protein interactions. Experimentally determined RBP binding sites are available in published enhanced cross-linking and immunoprecipitation (eCLIP) databases, such as the ENCORE dataset in ENCODE [85–87]. In the ENCORE dataset, *MCL1* has many RBPs bound to intronic and exonic regions, including SRSF1, which is known to affect *MCL1-S* to *MCL1-L* isoform ratios [88] (Figure 2H, boxes). The ENCODE SRSF1 binding site overlaps with a hotspot of RBP binding where 15 additional RBPs have been identified at the same position. The structures of hotspot elements may influence competitive RBP binding. Studies of *MCL1* suggest that hnRNPF/H regulates *MCL1* splicing and binds within intron 2 [89] (Figure 2H, boxed). However, each individual study is limited to select tissues and time-points. *SMN2* is a neural factor and is not expressed in the ENCODE project cell lines. Only one RBP, U2AF2 is significantly associated with *SMN2* in this dataset. However, experimental studies on *SMN2* have been performed in multiple laboratories suggesting that more than 40 RBPs bind pre-processed *SMN2* RNA around exon 7 and influence its splicing, reviewed in [90]. For example, the RBP TIA1 promotes *SMN2* exon 7 inclusion by binding with intron 7 and recruiting the U1 complex in proximity to the 5′ splice site [91]. TIA1 binding overlaps with the intronic TSL3 structure (Figure 2D, boxed). Antisense oligonucleotides targeting *SMN2* near the TIA1 site are predicted to open TSL3, make the TIA1 binding site more accessible, and promote exon 7 inclusion [72]. Predicting RBP sites is another option for genes that are less studied than *SMN2* but not expressed in commonly used cell lines [92]. As we learn more about the preference of different RBPs for sequence and structural features we will be able to better predict the impact of RNA structures within RBP-binding sites. Since protein binding sites can be influenced by RNA structure, they are important regions for structural studies.

**Figure 3:**
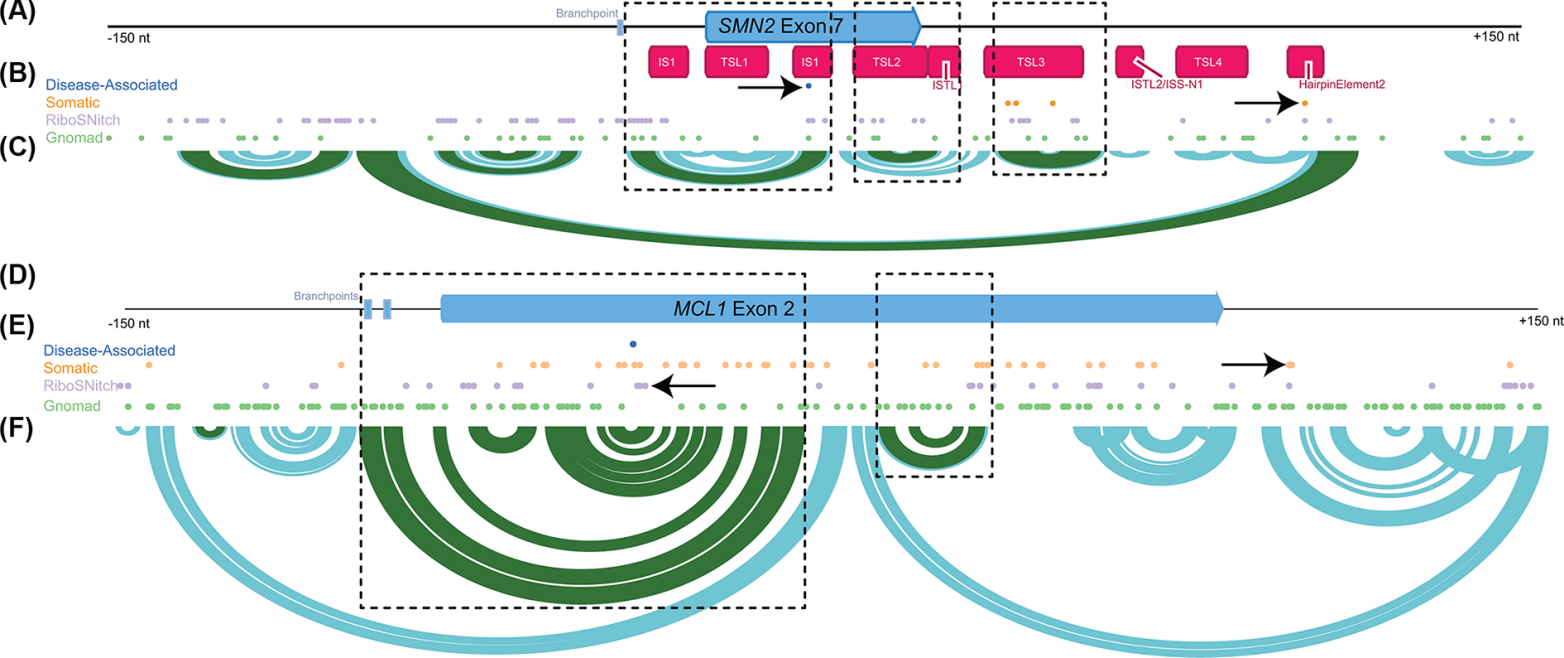
Identifying potential functional structures in *MCL1* RNA using genomic variation and RNA structure models (**A**) Schematic of human *SMN2* exon 7 surrounded by 150 intronic nucleotides on both sides. Known RNA structures in *SMN2* are annotated (red). (**B**) Genomic variants in *SMN2* from a variety of sources including variants that are disease-associated (blue), somatic (orange), predicted to change RNA structure (riboSNitches, purple) and inherited (green). The ClinVar disease-associated variant in SMN2 is a riboSNitch (arrow). Likewise, a somatic mutation in Hairpin Element 2 is a riboSNitch (arrow). (**C**) Arc diagram of the *SMN2* RNA structure generated with published SHAPE-MaP data [204] showing highly probable base pairing (>80%, green) and moderately probable base pairing (30–80%, blue). Predicted structures overlap with published structural elements (boxes). (**D**) Schematic of human *MCL1* exon 2 surrounded by 150 intronic nucleotides on both sides. (**E**) Genomic variation in the *MCL1* exon 2 region, colored as indicated above. RiboSNitches in proximity to the ClinVar disease-associated variant overlap with somatic mutations (arrow). We also highlight a riboSNitch that is a somatic mutation (arrow). (**F**) *MCL1* precursor RNA structure base-pairing probabilities generated from *in vitro* 5NIA SHAPE-MaP data and analyzed with shapemapper2 [205]. For both RNAs variants were retrieved from gnomAD [99], COSMIC [98], RNAsnp screening [102], and ClinVar [93]. RNA structures were generated with the RNAStructure package functions (partition and ProbabilityPlot [206]) and visualized in IGV [207].

Another way to identify functional regulatory elements that may affect RNA processing is to map disease-associated variants onto the RNA, such as variants found through GWAS and familial studies and in databases such as ClinVar and HGMD [93,94]. Additional variant-based approaches can use quantitative trait loci (QTLs), which are variants associated with a variety of phenotypes like expression (eQTLs), splicing (sQTLs) and alternative polyadenylation (apaQTLs) [95–97]. However, there are generally few disease-associated variants mapped to genes, particularly in non-coding regions such as introns. In *MCL1* and *SMN2* there are only a handful of disease-associated variants (Figure 3B,E). Rare variants and somatic mutations may also be informative for identifying functional regions of an RNA [98,99]. To directly connect variants with RNA structure elements there are computational models to estimate the impact of a variant on local and global RNA structure [100–103], reviewed in [104]. Variants that change RNA structure are relatively common and are called riboSNitches [3,100,105], reviewed in [106]. We find that across *SMN2* there are 95 riboSNitches, while in *MCL1* there are 40 riboSNitches [102] (Figure 3B,E). RiboSNitches that overlap with rare or phenotypic variants are candidates that indicate functional structures that may be involved in phenotypic differences. In *SMN2* a disease-associated variant found in ClinVar is also predicted to be a riboSNitch (Figure 3B, arrow). This riboSNitch falls within IS1, which is known to have structure that regulates *SMN2* splicing [70]. Another riboSNitch in *SMN2* overlaps with a somatic variant from COSMIC in the regulatory *SMN2* intron 7 hairpin element 2 (Figure 3B, arrow) [74]. Similar somatic variants are predicted to be riboSNitches in *MCL1*, including a riboSNitch in close proximity to a ClinVar variant (Figure 3E, arrows).

Finally, rather than starting from functional elements and analyzing whether RNA structure may impact that function, we can also start from experimental or predicted structural models. There are many webservers and software packages that can predict minimum free energy models or base-pairing probabilities with reasonably high expected accuracy [21–26]. Within these structural models, highly probable hairpin/stem-loop motifs are common regulatory elements, reviewed in [107]. In *SMN2*, selection of strong hairpin elements from an experimentally based structural model for further study would have identified internal stem 1 (IS1), TSL2 and TSL3, all of which have been shown to structurally influence *SMN2* splicing (Figure 3C) [55,70]. These elements are predicted despite the limited region selected for folding. In addition to these known structures in *SMN2* there are two highly probably hairpin motifs within *SMN2* intron 6 that could influence processing of *SMN2* precursor RNA (Figure 3C). Structural models of *SMN2* have been used by others to predict novel functional elements in *SMN2* [73]. *MCL1* precursor RNA also has highly probable hairpin and nested hairpin motifs in an experimentally based structural model (Figure 3F). In the nested hairpin, base-pairing is predicted between the branchpoint region and exonic sequence suggesting that this structure could affect 3′ splice site recognition and contribute to regulatory processing of *MCL1* RNA.

Currently it is difficult to identify functional structures within a precursor RNA. *SMN2* RNA is a well-studied model that shows the ability of conservation data, RBP binding site analysis, variant mapping and structural models to identify RNA structures that many influence *SMN2* splicing. We also highlight regions within the understudied *MCL1* RNA that may be of interest structurally to understand *MCL1* splicing. As only a handful of human genes have clear precursor RNA structural annotation, the lack of known functional structures even in a highly regulated gene like *MCL1* is unsurprising. The field will continue to acquire better secondary and tertiary structure models for *SMN2* and *MCL1* as new technologies for RNA structure modeling emerge, such as chemical probing techniques (Table 1) and cryo-EM. Although individual gene annotation for functional RNA structures is slow, an important goal is to identify enough functional structures in nascent RNAs to accurately predict functional structures directly from sequence.

## Mechanisms underlying structural recognition and dynamics

### How do precursor RNA structures influence RNA-binding protein interactions?

Proteins are principal effectors of cellular function. Proteins that interact with RNA are common and many studies have explored the sequence and structural preferences of RBPs [108–111]. The interaction between RBPs and RNA can be understood as a modular interaction between RNA-binding domains (RBDs) in the protein and target RNA elements. There are 16 well known RBDs, and likely additional non-canonical domains, reviewed in [112]. RBDs can be repeated or varied within a protein and the spacing between RBDs can influence how the protein interacts with its target RNA. Interactions between RBDs and RNA elements are governed by basic physical principles: hydrogen bonding, electrostatics, and base-stacking, reviewed in [113]. All three of these mechanisms are used by most RBDs for structure and sequence specific interactions. Most RBPs have degenerate sequence preferences that favor structurally accessible RNA sequences [108]. In general, sequence-specific interactions between RBPs and RNA occur through hydrogen bonding. Hydrogen bonds cannot readily form when the target nucleotides are base paired. However, even though most RBPs target unpaired nucleotides for sequence specificity, structural context does influence RBP interaction. This allows RBPs to differentiate between RNA binding elements even when sequence motifs are degenerate or similar to other RBP binding sites [108].

**Figure 4:**
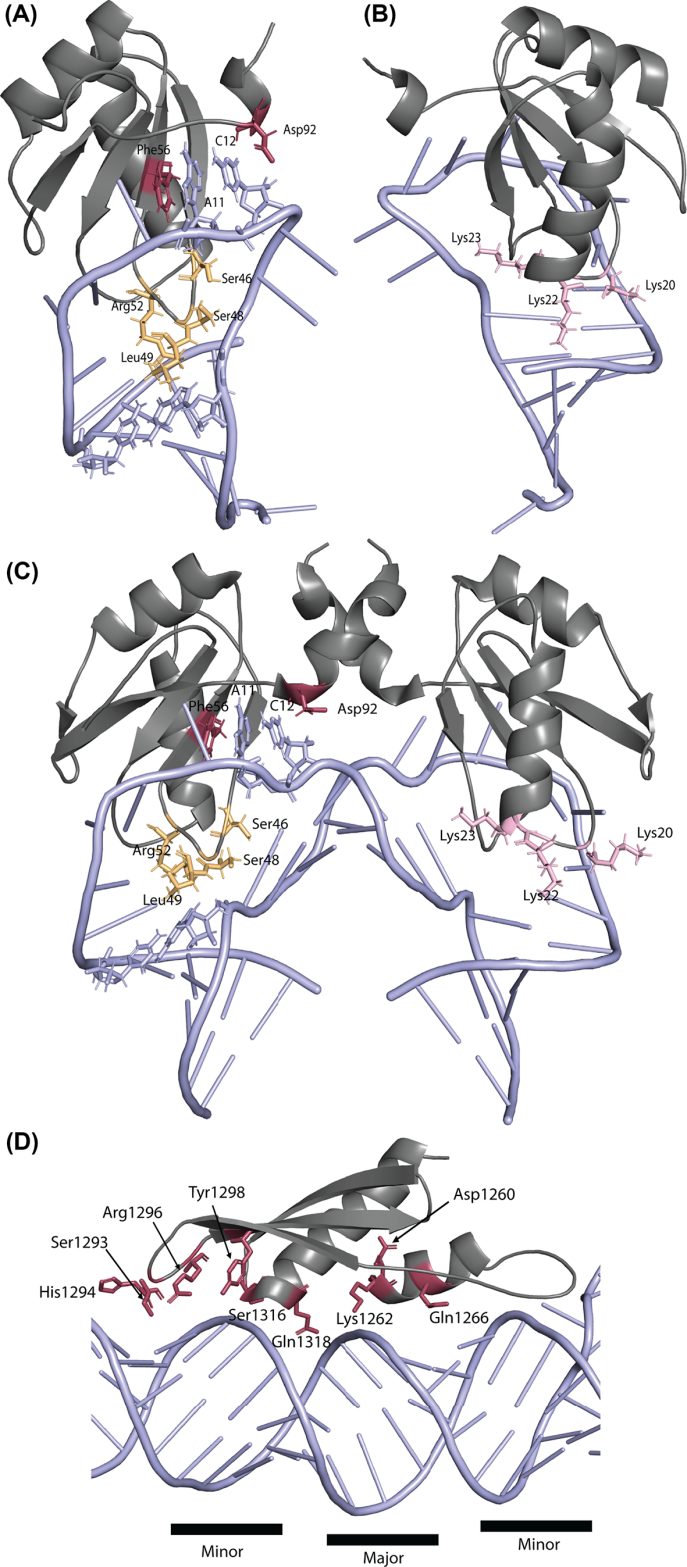
RNA interactions with RBPs (**A**) The RNA-binding domain of U1A snRNP protein (gray) binds the U1 snRNA stem loop II (blue). Base stacking of A11 and C12 between amino acids Phe56 and Asp92 (maroon). Amino acids Ser46, Ser48, Leu49, and Arg52 (light orange) lock the protein into the hole defined by the RNA structure and interact with bases C11-G16. (PDB 1URN [115]). (**B**) U1A amino acids Lys20 and Lys22 contribute electrostatic interactions that stabilize the phosphodiester backbone of the RNA. Lys23 interacts with the U1A protein loop located in the open RNA (pink) (PDB 1URN [115]). (**C**) U1A RMM dimer binds PIE RNA structure. Amino acids are highlighted in the same colors as outlined above (PDB 1DZ5, [121]). (**D**) DROSHA dsRBD (PDB 6V5B [128]) in complex with pri-miR-16-2. Amino acids Ser1293, His1294, and Arg1296 interact with ribose in the minor groove and Tyr1298 interacts with the minor groove phosphate backbone. Gln1318 electrostatically interacts with the phosphate backbone of the major groove [127].

One of the most common types of RBD is the RNA recognition motif (RRM). The U1A/SNF/U2A″ family is an example of RRM-containing proteins that can recognize structured RNA [114]. U1A is part of the spliceosome U1 snRNP. It binds specifically to the U1 stem-loop II (SLII) through structure specific base-stacking (Figure 4A, maroon), electrostatic interactions (Figure 4B, pink) and sequence-specific hydrogen bonding interactions (Figure 4A, orange) [115,116]. U1A binding is very specific and discriminates between stem-loops in U1 and U2 RNAs [117]. Despite this specificity, U1A is also capable of binding PIE RNA elements in its own 3′UTR and several other RNAs, including *SMN2*, to regulate polyadenylation [118,119] (Table 2). PIE elements are similar to the U1 SLII at the structure and sequence levels. Within a PIE element, duplicated stem-loops dimerize U1A (Figure 4C) and directly interact with polyA polymerase [120–122]. Although U2B contains a leucine-rich RBD rather than an RRM like U1A, it has a similar multifunctional ability. U2B interacts with the U2 RNA stem-loop IV with both structure and sequence specificity. However, in cancer cells, U2B has an extra-spliceosomal function wherein U2B binds intronic stem-loop structures and promotes splicing of cassette exons [61].

Double-stranded RNA-binding domains (dsRBDs) generally have strong structural preferences for their target RNAs. The cleavage factors Drosha and Dicer both contain dsRBDs and interact with precursor microRNAs through structure-dependent mechanisms [123,124]. MicroRNAs are processed from precursor RNAs (pri-miRNAs) that form extended stem-loops and are cleaved into pre-microRNAs and finally mature miRNAs. Pri-miRNAs interact with the microprocessor complex containing Drosha based on their helical RNA structure [125,126]. Drosha-dsRNA interactions are mediated by electrostatic interactions between the dsRBD (Figure 4D, gray) and two minor grooves, with minimal major groove interactions [127,128]. The extended stem-loop structure of pri-miRNAs is recognized at the transition between unpaired and paired segments at the bottom and top of the stem region. Flexibility in the pri-miRNA stem alters Drosha cleavage, ultimately resulting in the production of isoforms of mature miRNAs that can target different transcripts for translational repression [123]. The RBP Drosha and its partner DGCR8 recognize these structural junctions and structural changes in pri-miRNA, partially mediated by DDX3X, that result in different Drosha processing [123]. In addition to structure-specific recognition, Drosha also recognizes specific nucleotide sequences that are important for processing of pri-miRNAs. After Drosha processing, Dicer cleaves pre-miRNAs into duplex miRNAs. Dicer also recognizes structural elements of the pre-miRNA to discriminate between pre-miRNAs and other classes of RNA to cleave true pre-miRNAs into mature miRNAs [124].

While many RBPs have been extensively studied and their specificity determined, there are more than 2000 predicted RBPs in the human genome, and many ‘moon-lighting’ proteins with unrecognized RNA binding potential, reviewed in [112,113]. High-throughput studies to broadly characterize RNA-binding protein characteristics demonstrate the importance of both sequence and structural recognition [108,109]. Although the majority of RBPs prefer unpaired sequence motifs, they display varying sensitivity to structure. For example, RBPs including RBM22, RBM6, PRR3 and BOLL display preferences for motif-based partial pairing or a motif surrounded by paired nucleotides [108]. The little-known RBP ZNF326 even prefers sequence-based recognition within a completely paired structural motif [108]. By varying their ability to recognize structured sequences, RBPs effectively create a binding preference by incorporating the surrounding structural context of short sequence motifs [108]. Additional studies on the binding preferences of RBPs can help determine how RBPs recognize RNA and regulate gene expression.

### How is precursor RNA structure modulated in the cell?

While the sequence of an RNA is the primary input for many computational structure models, biological regulation affects the structure of RNA in the cell. One mechanism of regulation is the speed of transcription. Slow transcription by RNA pol II can influence the folding of known RNA structures, such as the hairpin structure at the 3′ end of histone transcripts [129]. Overall, slow transcription speeds increase base-pairing in nascent RNAs and lead to more efficient splicing [12]. The speed of RNA polymerase II transcription is a function of modifications to its carboxy-terminal domain; these modifications can be influenced by many biological processes, reviewed in [130,131]. Cell signaling by the myc pathway influences RNA pol II elongation speed, suggesting that RNA structure and processing can be globally altered under certain conditions, reviewed in [130,132]. In addition to global changes, individual gene loci may be prone to fast or slow transcription speeds based on their DNA and chromatin composition. For example, DNA G-quadruplexes influence transcription speed [133]. Additionally, several DNA- and RNA-binding proteins influence RNA pol II modification and elongation speed [134]. Each of these factors can be regulated by other cellular pathways, potentially making transcription speed a dynamic factor regulated globally and fine-tuned at individual loci. More research is required to explain how perturbation of transcription speed influences the ensemble of structures formed by a nascent transcript and how a structural ensemble may influence processing of the precursor transcript and its downstream output.

Nucleotide modifications are another cellular mechanism that can impact RNA structure. There are about 180 known types of RNA nucleotide modifications [135]. Two common modifications are methyladenosine (m6A) and pseudouridine (pseudoU), reviewed by [136,137]. Methylation of adenosine at the 6th position makes the residue more likely to be unpaired, resulting in changes to RNA structure than can affect RBP interactions [138,139]. Modification of uridine to pseudoU stiffens the RNA backbone, usually resulting in more stable RNA structure, but the impact on structure is dependent on the context of the pseudoU modification [140]. Lack of pseudoU modification is associated with altered structural dynamics in the ribosome, specifically in the way the sections of the ribosome rotate with respect to one another [141]. The ability of pseudoU to change the structure of the ribosome and modulate its dynamics probably contributes to the translational defect in cells with ribosomes that lack pseudoU [141]. Although modifications are the rule for noncoding RNAs such as ribosomal RNA and tRNAs, RNA modifications are also present in pre-processed mRNAs, reviewed in [137]. For example, both m6A and pseudoU are common in precursor RNAs and affect splicing [142–144]. Updates to computational modeling algorithms are beginning to consider the effect of m6A on the biophysical characteristics of structure folding [24]. The growing volume of functional RNA modifications suggests that these modifications constitute a dynamic cellular mechanism that regulates RNA structure.

RNA-binding helicases can change RNA structures in the cell. At least eight helicases, including DEAH-box helicases DHX19, DHX38, DHX8 and DHX15, are essential for splicing in human cells, reviewed in [145,146]. These helicases primarily assist with the release of splicing factors and precursor RNA at different stages of the splicing cycle. In addition, several helicases recognize stalled or improper splicing and are associated with degradation of these precursor RNAs [145]. In precursor microRNAs (pri-miRNAs), the DEAD-box helicase DDX3X impacts the structural flexibility of the pri-miRNA and influences alternative processing by Drosha [123]. DDX3X binds double-stranded RNA as a dimer, with one DDX3X primarily interacting with one RNA strand [147]. DDX3X regulation of pri-miRNAs results in differences in mature miRNA isoform composition across tissues and between normal and cancerous samples [123]. There are at least 64 human RNA helicases, primarily identified by homology to DEAD and DEAH helicases [148]. These helicases and other RBPs can alter RNA structure by mechanisms other than conventional helicase unwinding, reviewed in [112]. The interaction between helicases and RNAs is often dependent on the modification state of the helicase, allowing for dynamic regulation of RNA:protein interaction [149]. Additional research is needed to understand the function and regulation of helicases.

There is evidence that RNA structure is altered at different stages of an RNA molecule’s life cycle. Liu et al. found major differences between nuclear and cytoplasmic RNA structures in *Arabidopsis*, which suggested that RNA structure changed significantly from the precursor RNA to the mature transcript [10]. Structures in 3′UTRs can vary during different stages of development in zebrafish [11]. Differences in structure between RNAs *in vivo* versus purified from cells have been documented in yeast [4]. These studies demonstrate that RNA structure is not static. It is not clear in humans how structure is altered during processing of particular transcripts and whether refolding is a general characteristic that alters structure in predictable ways. Because RNA structure guides interactions with regulatory RBPs and nucleic acids, refolding of transcripts during processing has downstream implications for the fate of the transcript and gene expression.

### What is the influence of precursor RNA structure on gene expression?

**Figure 5:**
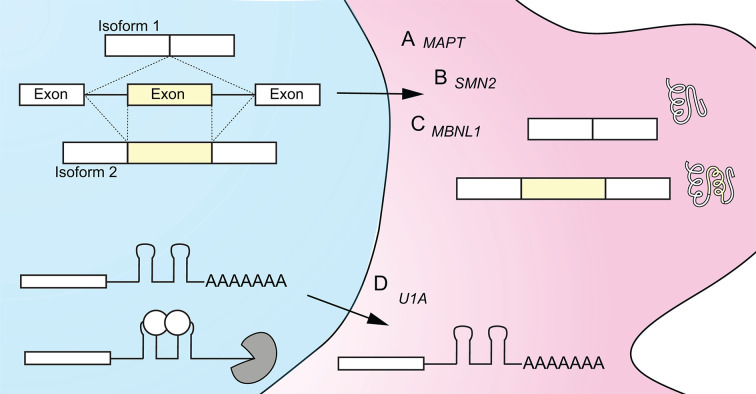
RNA structure impacts precursor RNA processing and influences gene expression Schematic showing RNA processing in the nucleus (left, blue) and the impact of processing on gene expression in the cytoplasm (right, pink). (**A**) Alternative splicing can result in either exon skipping or exon inclusion (left, top). In *MAPT* RNA, exon skipping is promoted by hairpin formation at the 5′ splice site of exon 10. Exon skipping produces 3R and 4R transcripts and their corresponding protein isoforms. The 3R and 4R MAPT proteins have different biological functions. (**B**) MBNL1 binding to *MBNL1* RNA at the 3′ splice site of intron 4 promotes exon skipping. Exon skipping produces a transcript missing a bipartite nuclear localization motif and a cell-wide protein isoform. Exon inclusion produces a transcript that is translated into a nuclear MBNL1 protein isoform. (**C**) In *SMN2* RNA, exon skipping is promoted by hairpin formation at the 5′ splice site of exon 7. Exon skipping results in a protein isoform of SMN that is less stable than the full length SMN containing exon 7. Levels of SMN are associated with the severity of spinal muscular atrophy. (**D**) Most RNAs are polyadenylated at the 3′UTR (bottom). In *SMN2* processing the 3′ end of the transcript contains a PIE structural element bound by U1A that inhibits polyadenylation. Little or no polyadenylation leads to transcript instability and a decrease in RNA levels.

In this review we have discussed multiple examples of how RNA structures in human precursor RNAs impact RNA processing and alluded to the effect of altered processing on subsequent gene expression. One impact of altered splicing is production of an alternative protein isoform (Figure 5A–C). In *MAPT*, when exon 10 is skipped the mature transcript produces a Tau protein with three rather than four microtubule binding domains (Figure 5A) [48]. These Tau protein isoforms have different biological activities and altering their ratio is correlated with development of frontotemporal dementia and other neurodegenerative diseases [48,49]. In *SMN2, e*xon 7 is normally skipped, resulting in an unstable SMN protein isoform (Figure 5B) [150]. Spinal muscle atrophy occurs when neither *SMN1* nor *SMN2* can produce stable SMN protein. Exon 5 skipping in *MBNL1* RNA results in loss of part of the bipartite nuclear localization element in MBNL1 protein and cell-wide localization rather than nuclear localization [151–153] (Figure 5C). The sequestration of MBNL1 in toxic repeats is an important factor in myotonic dystrophy [154]. RNA degradation can also be influenced by RNA structures that affect processing. For example, *U1A* precursor RNAs contains a PIE element that is bound by two U1A proteins [118]. Binding of U1A to its own transcript results in inhibition of polyadenylation and a decrease in *U1A* RNA [118,155] (Figure 5D). PIE elements in *SMN2* and other transcripts also can be bound by U1A and inhibit polyadenylation, ultimately resulting in lower levels of RNA [118,119]. Altered RNA processing can also influence nonsense-mediated decay [156], RNA localization [157] and protein expression [158]. Nascent RNA structure has ripple effects on all aspects of the RNA life cycle and can contribute to human diseases.

### How can precursor RNA structure be targeted by therapeutics?

Because RNA structure influences its functional interactions with other molecules, structure is a target for intervention, including at the nascent RNA stage. Antisense oligonucleotides (ASOs) can be designed to alter RNA structure, reviewed in [159,160]. In a structured RNA, bases normally interact in *cis* to form standard hairpins or stem-loop structures. An ASO can compete for base pairing in *trans*. The hybridization between the ASO and its target RNA opens up nucleobases for interaction with other nucleotides or proteins and could have global effects on structure. In the 7SK snRNP structural rearrangement is important for release of kinases involved in phosphorylation of the Poll II carboxy-terminal domain, leading to transcriptional control. ASOs that target sequences within 7SK dynamic hairpins block the structural transition of 7SK from one state to another and alter the ability of 7SK to regulate transcription [161]. The SARS-CoV2 corona virus has a highly structured single-stranded RNA genome [162]. One strategy currently under development for treatment of SARS infection is an ASO designed to disrupt a 3′ stem-loop involved in viral replication, reviewed in [163]. A similar mechanism of competing hybridization has been used to develop toehold switches, which switch structures in the presence of a particular RNA sequence to allow translation [164]. Toehold sensors have been developed for many applications including as a method to detect viral infections like SARS-CoV2 and Zika [165,166] and identify genomic variation [167]. There is software available to design toehold structures (NUPACK) [168]. Other hybridization methods that impact RNA structure have been developed to target transcriptional regulation [169].

Although hybridization offers a straightforward mechanism of structure change, ASOs are difficult to deliver to human tissue, whereas small molecules are generally more tractable for medical treatment. Small molecules can target and stabilize or destabilize specific RNA structures, reviewed in [170–172]. The capacity of small molecules to act on RNA structures was evident early on from bacterial riboswitches, which are designed to recognize a variety of different small molecules (e.g., metabolites) and change conformation to effect transcriptional or translational regulation, reviewed in [173]. Although most proof-of-principle molecules target noncoding or viral RNAs [174,175], small molecules that target RNA structures can be used to control nascent RNA processing. The FDA approved small molecule risdiplam has been developed to target *SMN2* splicing at exon 7, reviewed in [176]. Although the exact mechanism of action is still under investigation, these molecules may function to stabilize the interaction between the U1 spliceosome and the 5′ splice site [177,178]. Modulating RNA structure to diagnosis or to treat disease is a rapidly growing field. Targeting function RNA structures in precursor RNAs is an important direction for therapeutic development.

## Summary

RNA molecules are naturally structured. Due to the low abundance, long-length, and flexible nature of nascent RNAs, precursor RNA structure is understudied. New structural methods are continuing to advance our technological capabilities and document structures within precursor RNAs. In particular, chemical probing and cryo-EM methods have expanded our understanding of secondary and tertiary structures. However, even when structural models are available, it is difficult to identify functional structures and understand their mechanisms. Structures within precursor RNAs determine how nascent transcripts interact with protein and nucleic acid co-factors. By influencing these interactions, RNA structure influences RNA processing pathways. Most studies have focused on the impact of structure on splicing and polyadenylation, but future research may tell us more about how RNA structure affects other processing pathways like RNA editing. Due to their impact on processing, RNA structures impact gene expression and play a role in disease. We are beginning to develop antisense oligonucleotides and small molecule methods to alter RNA structure *in vivo*; these methods can be broadly applied to target functional RNA structures, including those that regulate RNA processing.

## Abbreviations

ASO: antisense oligonucleotide
dsRBD: double-stranded RNA-binding domain
eCLIP: enhanced cross-linking and immunoprecipitation
RBD: RNA-binding domain
RNP: Ribonucleoprotein
RRM: RNA recognition motif
